# Survival Analysis of F98 Glioma Rat Cells Following Minibeam or Broad-Beam Synchrotron Radiation Therapy

**DOI:** 10.1186/1748-717X-6-37

**Published:** 2011-04-13

**Authors:** Silvia Gil, Sukhéna Sarun, Albert Biete, Yolanda Prezado, Manel Sabés

**Affiliations:** 1 Centre d'Estudis en Biofísica, Faculty of Medicine, Autonomous University of Barcelona, Cerdanyola del Vallès, Spain; 2 European Synchrotron Radiation Facility (ESRF), Grenoble, France; 3 Hospital Clínic, University of Barcelona, Barcelona, Spain

## Abstract

**Background:**

In the quest of a curative radiotherapy treatment for gliomas new delivery modes are being explored. At the Biomedical Beamline of the European Synchrotron Radiation Facility (ESRF), a new spatially-fractionated technique, called Minibeam Radiation Therapy (MBRT) is under development. The aim of this work is to compare the effectiveness of MBRT and broad-beam (BB) synchrotron radiation to treat F98 glioma rat cells. A dose escalation study was performed in order to delimit the range of doses where a therapeutic effect could be expected. These results will help in the design and optimization of the forthcoming in vivo studies at the ESRF.

**Methods:**

Two hundred thousand F98 cells were seeded per well in 24-well plates, and incubated for 48 hours before being irradiated with spatially fractionated and seamless synchrotron x-rays at several doses. The percentage of each cell population (alive, early apoptotic and dead cells, where either late apoptotic as necrotic cells are included) was assessed by flow cytometry 48 hours after irradiation, whereas the metabolic activity of surviving cells was analyzed on days 3, 4, and 9 post-irradiation by using QBlue test.

**Results:**

The endpoint (or threshold dose from which an important enhancement in the effectiveness of both radiation treatments is achieved) obtained by flow cytometry could be established just before 12 Gy in the two irradiation schemes, whilst the endpoints assessed by the QBlue reagent, taking into account the cell recovery, were set around 18 Gy in both cases. In addition, flow cytometric analysis pointed at a larger effectiveness for minibeams, due to the higher proportion of early apoptotic cells.

**Conclusions:**

When the valley doses in MBRT equal the dose deposited in the BB scheme, similar cell survival ratio and cell recovery were observed. However, a significant increase in the number of early apoptotic cells were found 48 hours after the minibeam radiation in comparison with the seamless mode.

## Background

Gliomas are among the most frequent primary brain tumors in adults, with an incidence of approximately 5/100,000 among the general population [1], and despite significant advances in cancer therapy, treatment of high-grade gliomas is only palliative.

A radical radiotherapy treatment of radioresistant tumors would require the development of new techniques allowing to spare the sensitive surrounding normal tissue.

Since 1990s synchrotron radiation has become one of the most valuable tools in experimental radiotherapy in the quest for a radical treatment for gliomas. Synchrotron sources are ideal for spatially fractionated techniques such as Microbeam Radiation Therapy (MRT) and Minibeam Radiation Therapy (MBRT), currently under development at the European Synchrotron Radiation Facility -ESRF- in Grenoble, France. The reason is that synchrotron beams possess two relevant features: a negligible divergence allowing to have sharp defined irradiation edges, and a 10^6 ^times higher fluence of x-rays than standard medical irradiators, which permits to avoid the beam smearing to the cardiosynchronous pulsations [2].

These two innovative techniques, MRT and MBRT, are based on the dose-volume effect: the smaller the irradiated volume is, the higher the dose tolerances of the healthy tissue are [3]. The beam width ranges from 25 to 100 μm in MRT, whereas in MBRT beams of 500 - 700 μm width are employed. That is to say, one or two orders of magnitude thinner than the ones used in conventional radiotherapy. The energy spectrum employed ranges from 50 to 500 keV, and with a mean energy at around 100 keV [4].

The dose is spatially fractionated: high doses are delivered in one fraction by using arrays of intense parallel beams. The interbeam separation is 200 μm or 400 μm in the case of MRT and 600 μm in MBRT. The dose profiles consist of peak and valleys, with high doses in the beams paths and low doses in the spaces between them [5].

During the last two decades many in vivo experiments have shown the sparing effect provided by MRT in the healthy tissue of the central nervous system (CNS) [6-10]. The spatial fractionation of the dose would provide a further gain in tissue sparing due to a biological repair of the microscopic lesions by the minimally irradiated contiguous cells [6,11].

In parallel it was observed that the tumor area is irreversibly damaged by the extremely high doses deposited on it [8,11,12] by using microbeams.

The thin microbeams (and their associated small beam spacing) need high dose rates, only available at synchrotrons nowadays. This limits their widespread clinical implementation. In addition, the high lateral scattering produced by beam energies higher than 200 keV would lead to the loose of the healthy tissue sparing [5]. The requirement of low-energy beams limit the dose penetration to the tissue.

To overcome those drawbacks MBRT has been recently proposed by Dilmanian et al. [13], also based on the dose-volume effect explained before. The increase of the thickness of the beams up to 0.68 mm, with a center-to-center (c-t-c) distance between them corresponding to the double of this value, might result in some advantages over the MRT such as [14]: i) The dose profiles of minibeams are not as vulnerable as those of microbeams to beam smearing from cardiosynchronous brain tissue pulsation [2]. Hence the high dose rate of synchrotron sources is not needed, and it makes feasible their forthcoming clinical implementation with proper technical improvements. ii) In MBRT, the use of higher beam energy is feasible [5] resulting in a lower entrance dose to deposit the same integrated dose within the tumor with respect to MRT.

In a first in vivo experiment performed by Dilmanian and coworkers [13], minibeams as thick as 0.68 mm seemed to keep (part) of the sparing effect observed in MRT, supporting a potential application of minibeams to treat tumors with minimal damage to the surrounding healthy tissues.

For the aforementioned reasons, MBRT has been recently implemented at the ESRF ID17 Biomedical Beamline [14].

This work is framed in first studies of the effectiveness of MBRT for the treatment of gliomas. Although the results of in vitro studies cannot replace in vivo works, they should be performed a priori in order to design better animal experiments, and delimit to some extent the range of doses where the therapeutic window for a new radiation treatment could be expected.

To the best of our knowledge, although until the moment there is a lack of published data concerning in vitro experiments with MBRT. Nevertheless, this kind of experiments based on cell cultures play an important role under the point of view of the ethical standards based on the so-called three Rs: *Reduce *the number of animals in each experiment, *Refine *the methodologies used on them, and *Replace *the animals for cells whenever possible in order to assess the endpoint of a radiation treatment, providing more information previously in vitro before to scale up this to a small animals. Moreover, cells studies are required to quantify the apoptotic events after the irradiations by using techniques such as flow cytometry, only feasible in in vitro experiments [15].

The main aim of this work was to assess the endpoint, or threshold dose from which an important enhancement in the effectiveness is achieved for both, minibeams and seamless irradiations on F98 cell cultures. Due to the fact that the ratio of post-irradiated living cells obtained from flow cytometric analises is not proportional to their recovery ability with time, the metabolic activity of this sort of cells at different days was also analyzed for each deposited dose and irradiation mode.

## Methods

### Cell line and culture conditions

In current experimental neuro-oncology many widespread cell rat models are used [16], being 9L gliosarcoma and F98 glioma the most extended cell lines [17,18].

The F98 rat glioma cell line was obtained from the American Type Culture Collection. This kind of tumor cells has an infiltrative pattern of growth within the brain when they are inoculated as a tumor model, and also a weakly immunogenic response. For these characteristics, F98 simulates human glioblastoma multiforme [16]. In the experiments, F98 cells were cultured in flasks of 25 cm^2 ^on Dulbecco's Modified Eagle's Medium (DMEM) supplemented with 1% penicillin/streptomycin, and with 10% fetal calf serum to reach confluence. Two hundred thousand F98 cells were seeded per well in 24-well plates and incubated for 48 hours at 37°C.

All the supernatants were removed and replaced by fresh medium just after irradiation.

### X-ray irradiations

Both minibeam and seamless in vitro irradiations were carried out at the ESRF ID17 biomedical beamline where the x-rays source consists in a wiggler with 15 cm of period, and a maximum magnetic field of 1.6 T. It is located 40 meters far from the sample. The dose rate at the target position is 5350 Gy s^-1 ^[14].

Fifty per cent of the previously seeded plates were irradiated vertically in front of the beam at different doses with one array of minibeams of 600 μm-width, with a center-to-center distance of 1200 μm, and covering a field of 2 × 2 cm^2^. The remaining plates were irradiated with a seamless beam configuration.

### Monte Carlo simulations and dose calculations

**Figure 1:**
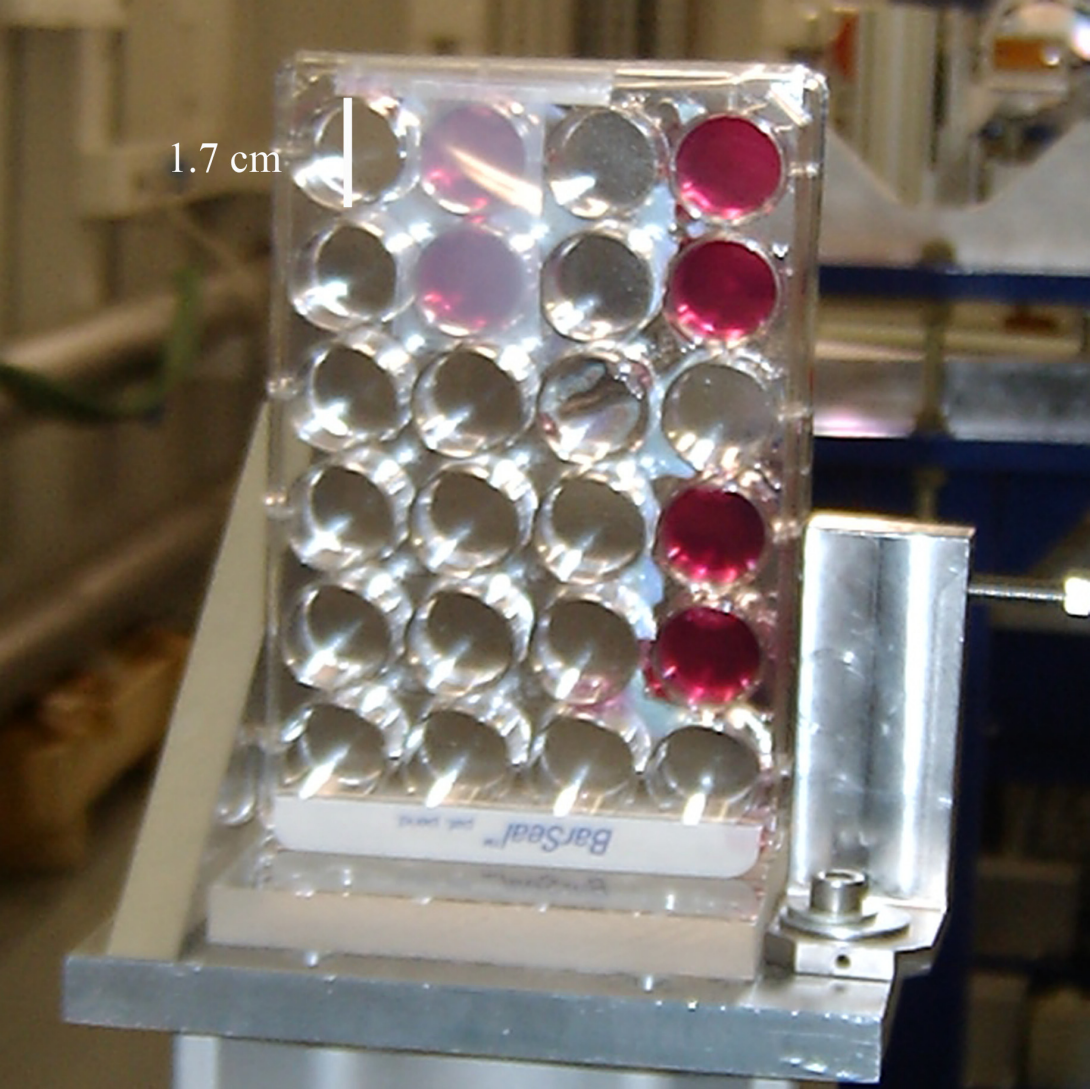
**24-well plate's picture (partially)**. Picture corresponding to a 24-well plate like those employed in the irradiations, either for MBRT or seamless irradiations. The verification of the dose assessed was done by using radiochromic films just above the wells as it is shown.

The dose deposited on the cells monolayer was assessed both theoretically (Monte Carlo simulations) and experimentally. In order to reduce an extra dose deposition on cells from the scattering of filled wells contiguously, the seeding well configuration for each plate was the same as those represented in Figure 1. Moreover, to have similar valley doses like the ones expected in the forthcoming in vivo experiments with small animals the wells were filled up with medium, remaining the cells on the bottom, at 1.7 cm depth. If the cell layer had been irradiated directly, in the absence of medium, the peak-to-valley dose ratio (PVDR) would have been much higher than the one calculated for the in vivo experiments with rats, due to reduced scattering filling the valleys [4].

Absolute dose at 1.7 cm depth was measured with an ionization chamber (PTW 31002 [http://www.ptw.de]) in a water-equivalent RW3 material (Goettingen White Water) with a seamless field of 2 × 2 cm^2^. The dose measured in this broad field configuration was converted to peak doses at 1.7 cm by using the phantom scatter factor. That factor was been assessed by using Monte Carlo simulations (PENELOPE 2006 [19]) and verified experimentally with radiochromic films (International Specialty Products, http://online1.ispcorp.com).

The plate geometry was modeled using the geometry package in PENELOPE. Monte Carlo simulations for peak and valley doses assessment were performed. Experimental validation of the calculations was carried out by using radiochromic films placed at different depths in the water-equivalent RW3 material phantom.

A dose escalation study was performed with both, MBRT and broad beam (BB).

Although some MRT studies indicate that the tumor control depends both on the peak and valley doses [8,20], this still needs to be confirmed in MBRT studies (not the scope of this work). In this first study the effectiveness of MBRT and BB irradiations was compared by taking MBRT valley doses in as a reference.

It was considered relevant to start assessing first which is the range of valley doses required to produce tumor ablation. Those results are expected to help designing the first in vivo experiment, the objective being the assessment of the dose tolerances of the rat brain. All this would allow delimiting the position of the possible therapeutic window for gliomas in this new radiotherapy approach (to be confirmed in in vivo studies) and its comparison with conventional methods.

In addition the higher the valley doses, the higher the density of lesions in the tumor. Therefore, it could be hypothesized that a higher tumor control probability is expected, to be confirmed in future animal studies.

Hence an escalation taking as a reference the valley dose seemed justified. Tables 1 and 2 report the doses in MBRT and BB respectively, evaluated in this work.

**Table 1:** Peak doses, valley doses, and integrated doses for minibeam irradiation:

Peak dose (Gy)	63	126	189	252	315	420
Valley dose (Gy)	6	12	18	24	30	40

Integrated dose (Gy)	34.5	69.0	103.5	138.0	172.5	230.0

The peak and valley doses (standard error, SE = ± 5%) delivered with MBRT. The integrated dose represents the mean dose deposited between the peak and the valley regions.

**Table 2:** Doses assessed by the seamless irradiation:

Dose (Gy)	6	12	18	24	30	40

Doses (standard error, SE = ± 5%) delivered with the seamless radiation. Note that these doses are the same than the valley doses in the case of minibeams.

The integrated dose was directly calculated as an average of each peak and valley doses (Table 1).

### Techniques used post-irradiation to assess cellular damage

#### Flow cytometry for the analysis and quantification of post-irradiated cell populations (alive, early apoptotic and dead cells)

This technique is based on detection of apoptosis-associated changes in distribution of an inner plasmatic membrane phospholipid called phosphatidylserine (PS). Early in apoptosis PS undergoes translocation to the external surface of the plasmatic membrane. By using the protein annexin V, which has a high affinity for the negatively charged PS, conjugated with a fluorochrome such as fluorescein isothiocyanate (FITC), it is possible to identify apoptosis in post-irradiated cells [15,21]. In order to detect also late apoptotic and necrotic cells, FITC-conjugated annexin V was used in combination with a DNA staining with propidium iodide (PI) (Annexin-V-FLUOS staining kit, Roche). Most of the flow cytometric methods do not allow for a clear distinction between late apoptotic and necrotic cells, due to in both cases, the dead cells are double-stained with annexin-V-FITC and PI.

To obtain the correct percentage of each cell population 48 hours after irradiation, it was essential to add the floating cells to the trypsinized ones and mesure all them (10^5 ^cells at least), before performing flow cytometry by using a FACS Calibur (Becton Dickinson). Moreover, cells of minibeam's peak and valley regions were analyzed together. The final data about non-apoptotic live cells (FITC-negative, PI-negative), early apoptotic cells (FITC-positive, PI-negative), and late apoptotic cells as well as necrotic (FITC-positive, PI-positive) were analyzed with WinMDI 2.9 free software (Scripps research instituteville, La Jolla CA, USA). The population was gated in order to eliminate debris.

#### QBlue assay for the metabolic evaluation of surviving cells

QBlue cell viability test (QBlue Cell Viability Assay kits, BioChain, USA) is based on the conversion of a non fluorescent reagent (resazurin) into a high fluorescent product (resorufin), upon mitochondrial reduction by the remaining living cells. To perform this test, the remaining living cells just after each irradiation were trypsinized and counted. In a 96-well plate, ten thousand cells were seeded per well and incubated at 37°C in order to check cell recovery at days three, four, and nine after irradiation, with a Wallac Fluorometry plate reader (excitation wavelength at 550 nm and emission at 615 nm).

Since the supernatants containing either early apoptotic as dead cells were not included, the metabolic activity measured was only due to the remaining living cells after irradiation.

Moreover, cells used to perform the QBlue test were irradiated in different 24-well plates than those analyzed by flow cytometry.

### Statistical evaluation of analyzed data

All flow cytometries were performed in triplicate, whereas the QBlue test was done up to eight times per dose and radiation mode. For both experimental techniques, dose-response data were evaluated using a standard two-factor analysis of variance (ANOVA) test in order to analyze the effect of the dose, radiation mode, as well as the interaction between them. Further comparisons among the 7 doses for each mode of irradiation were Bonferroni corrected using GraphPad Prism. A value of *p *> 0.05 was used to indicate no-significant differences (ns), whereas asterisks denote the following cutoff differences between the groups: *p < 0.05, **p < 0.001, ***p < 0.0001.

Results for control cells (without any radiation treatment) obtained from flow cytometries were fitted as having 100% of survival and 0% of both, death and early apoptotic indexes. The values for the rest of the cellular populations obtained from flow cytometric analyses were normalized to the control group.

## Results

In this section, results concerning the cell survival ratio analyzed by flow cytometry 48 hours following the treatments are presented first. In the second part, data obtained from the QBlue assays on days 3, 4, and 9 after both, MBRT and BB, are shown.

### Flow Cytometry data

**Figure 2:**
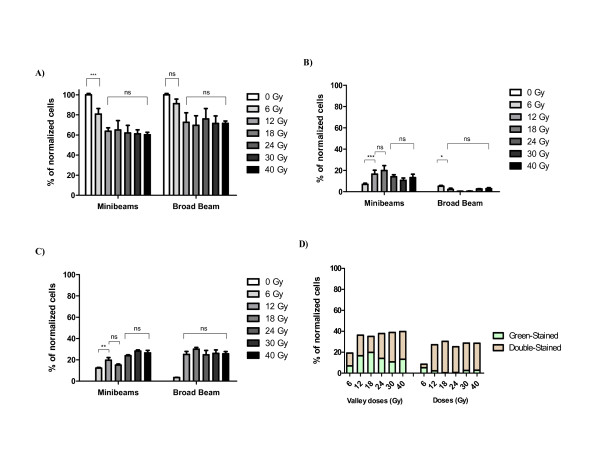
**(A-D) - Flow cytometry results**. Normalized histograms obtained by flow cytometry 48 hours after irradiation at several doses, with MBRT (left) and BB (right). The plots correspond to: (*A*) the unstained surviving cells, (*B*) the positive green fluorescence cells, corresponding to those cells undergoing apoptosis in an early stage, (*C*) double stained cells, with propidium iodide and annexin, indicating as late apoptotic as necrotic dead cells, and (*D*) the early (in green) and late (in green and red) apoptotic cells, in order to represent altogether the different death processes.

Normalized data for unstained surviving cells (Table 3), and plotted in histograms in Figure 2A, indicated the same dose-response for both kind of irradiations. Common observations are: i) a significant decrease in survival for cells irradiated up to 12 Gy, and ii) the same percentage of living cells for doses higher than 12 Gy (Table 3). For these data, the endpoint could be established around 12 Gy (by flow cytometry) after both irradiation modes. The same conclusion can be reached by evaluating altogether apoptotic and necrotic cells as a function of dose (Figure 2-D), where a significant increase in cell death is achieved from doses ≥ 12 Gy.

**Table 3:** Normalized values for surviving cells.

Seamless and valley doses (Gy)	% of normalized surviving cells after Minibeams (MBRT) (Mean ± SE)	% of normalized surviving cells after Broad Beam (BB) (Mean ± SE)
0	100	100
6	80.9 ± 5.6	91.2 ± 4.5
12	63.8 ± 3.3	72.7 ± 9.4
18	65.1 ± 9.2	69.6 ± 9.5
24	62.1 ± 7.5	76.0 ± 10.4
30	61.1 ± 4.0	71.4 ± 7.6
40	60.3 ± 2.4	71.5 ± 2.4

These values are represented in histograms in Figure 2*A*

Percentages of normalized values (mean percentage ± standard error) for surviving cells analyzed by flow cytometry 2 days after MBRT (valley doses) and BB. These data are graphically represented in histograms in Figure 2*A.*

However, one of the most interesting findings was the significant increase (*p *< 0.0001) of cells undergoing early apoptosis 48 hours after MBRT compared to BB (Figure 2B and Table 4). MBRT led to a clear increase in the number of pre-apoptotic cells up to a maximum reached at 12 Gy, remaining unchanged until 18 Gy. Only at higher valley doses (≥ 24 Gy) the percentage of pre-apoptotic cells decreased (Table 4), showing the preference of F98 cells of dying by necrosis (or late apoptosis) at those valley doses.

**Table 4:** Normalized values for early apoptotic cells.

Seamless and valley doses (Gy)	% of normalized early apoptotic cells after Minibeams (MBRT) (Mean ± SE)	% of normalized early apoptotic cells after Broad Beam (BB) (Mean ± SE)
0	0	0
6	7.0 ± 1.0	5.2 ± 0.8
12	16.6 ± 3.6	2.2 ± 1.1
18	19.9 ± 4.7	0.4 ± 0.1
24	14.1 ± 1.9	0.6 ± 0.2
30	10.7 ± 2.2	2.6 ± 0.3
40	13.3 ± 3.2	2.9 ± 0.9

These values are represented in histograms in Figure 2*B*.

Percentages of normalized values (mean percentage ± standard error) for early apoptotic cells analyzed by flow cytometry 2 days after MBRT (valley doses) and BB. These data are graphically represented in histograms in Figure 2*B*.

Figure 2C and Table 5 depicted the normalized percentage of cells undergoing late apoptosis and necrosis 48 hours after treatments. In the case of minibeams, a stepwise increase in the number of dead cells was observed up to a valley dose of 18 Gy. Note that in this case the percentage of dead cells at 12 Gy was practically the same (*p *> 0.05) than at 18 Gy (Table 5).

**Table 5:** Normalized values for dead cells.

Seamless and valley doses (Gy)	% of normalized dead cells after Minibeams (MBRT) (Mean ± SE)	% of normalized dead cells after Broad Beam (BB) (Mean ± SE)
0	0	0
6	12.2 ± 0.8	3.4 ± 0.2
12	19.7 ± 2.4	25.0 ± 2.9
18	15.1 ± 1.0	30.0 ± 1.7
24	23.8 ± 0.8	24.7 ± 4.1
30	28.2 ± 1.2	26.1 ± 3.3
40	26.5 ± 2.4	25.6 ± 2.2

These values are represented in histograms in Figure 2*C*.

Percentages of normalized values (mean percentage ± standard error) for dead cells, including late apoptotic and necrotic cells analyzed by flow cytometry 2 days after MBRT (valley doses) and BB. These data are graphically represented in histograms in Figure 2*C*.

No significant differences were observed for doses ranged from 24 Gy to 40 Gy, neither among them nor in comparison with dead cells irradiated between 12 Gy and 40 Gy with BB. Hence, the percentages of cell death at higher doses seemed not to significantly differ (*p *> 0.05) according the radiation feature.

Therefore, the lower percentage of living cells observed 48 hours after MBRT led to assess a higher effectiveness of minibeams against tumor cells (*p *= 0.0002), although for both irradiation schemes the endpoint analyzed by flow cytometry was established around 12 Gy.

In addition, the process of cell death clearly differed as a function of dose, as well as of the synchrotron radiation mode.

### Metabolic response analyzed by QBlue assay

The cellular recovery, analyzed as metabolic activity of living cells, was measured following a kinetic by using the non-toxic QBlue assay.

**Figure 3:**
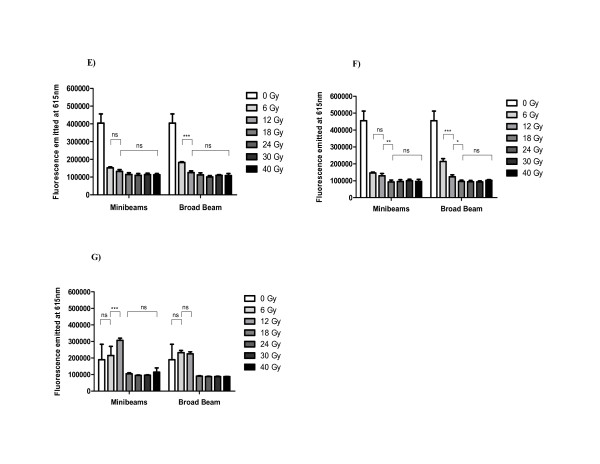
**(E-G) - Results from the QBlue tests**. Normalized histograms after synchrotron MBRT (left) and BB (right) at several doses. The plots correspond to QBlue tests performed on (*E*) day 3, (*F*) day 4, and (*G*) day 9 after radiations. The most significant cell recovery could be observed at the nineth day for doses ≤ 12 Gy.

The first measurement was performed at the third day after treatments, when the non-irradiated cells already showed a clear higher recovery than those irradiated either with minibeams or with seamless configuration (Figure 3E). Among the irradiated cells, the only significant increase of the metabolic activity was observed in cells irradiated with broad beam at 6 Gy (Figure 3E).

This larger cell recovery observed at the third day after BB irradiation was in agreement with the also higher percentage of surviving cells obtained by flow cytometry one day before.

The QBlue assay performed at the fourth day showed once more that the largest recovery ability was for those cells irradiated with broad beam at doses ≤ 12 Gy (Figure 3F).

Although cell recovery was larger after BB irradiation than those observed after MBRT, the endpoint for both irradiation modes was observed around 18 Gy. It was due to the lack of metabolic activity for doses ≥ 18 Gy in any mode of irradiation.

This result was also confirmed by the last QBlue measurement carried out on day nine (Figure 3G). Moreover, in this last test, a complete cell recovery was observed for cells irradiated at doses equal or lower than 12 Gy. Under these conditions, viable cells took profit of free space between them to reach confluence, leading to arbitrary variations in the measurement of their metabolic activity as those shown in Figure 3G.

Hence, thanks to this additional method of measuring viability, it was possible to check that for both synchrotron irradiations, doses as high as 18 Gy should be deposited on F98 cells to avoid their recovery with time.

## Discussion

Flow cytometry is widely used in studies of cell populations, because it provides information about both cell size and intracellular structure on each cell as the suspension passess at high speed through a laser beam [22]. Furthermore, it offers the unique possibility of multiparametric analysis, including identification and quantitation of apoptotic and necrotic cells after treatments.

Exploiting this technique, we could establish that the ratio of F98 glioma cells in early stages of apoptosis was larger when cells have been previously irradiated with MBRT than with BB. Considering that the analyses were performed two days after irradiation, this several-times larger increase in apoptotic index may either indicate that: i) there was a larger amount of cells dying by apoptosis by this time, compared to those seamless-irradiated, or ii) the same number of cells were dying, but the duration of apoptosis was more prolonged after MBRT [23].

The first hypothesis is more likely to be the right one, taking into account the considerable amount of published data [15,21,23] reporting that necrotic form of cell death is associated with acute cell injury, whereas apoptosis is gene directered, triggered in some cases as an adaptative response to the spatially fractionated cell irradiation [24-26].

By studying the nuclear cell morphology, Dilmanian et al. [24] detected in cell-culture microbeam studies that the mode of cell death following very-high-dose irradiations was by apoptotis. Nevertheless, those studies should be applied not only for healthy but also for tumor cells and further experiments are needed to clarify the point concerning the larger ratio of cells undergoing early apoptosis after MBRT.

On the other hand, two times higher valley doses (≥24 Gy ) were needed with MBRT to reach the same percentage of late apoptotic and necrotic cells than those irradiated with a broad-beam configuration. Despite this, cells irradiated with MBRT showed higher ratios of early apoptosis in any dose with respect to BB (Figure 2D). This fact indicates that MBRT provides a higher effectiveness in terms of cell killing, as observed in those plots corresponding to flow cytometries (see Results).

The endpoint measured in terms of cell survival could be established just before 12 Gy for both irradiation modes.

Nevertheless, the effectiveness on tumor cells of both, MBRT and BB irradiations, cannot be assessed exclusively by the death of cells after treatment analyzed by flow cytometry, and other biophysical aspects such as the recovery ability with time have also to be considered. For this purpose, a complementary technique to flow cytometry was used. It allows to measure the recovery of the remaining living cells after each irradiation. In contrast to others such as the (3-(4,5-dimethylthiazol-2-yl)-2,5-diphenyltetrazolium bromide (MTT), QBlue test provides an adequate estimation of cell proliferation, keeping the cells alive. In this way, a kinetic study can be performed. In addition, Alamar QBlue is more sensitive than MTT [27].

The results from the second technique points at around 18 Gy the endpoint for MBRT and BB. This endpoint corresponds to the dose from which there was not observed any cell recovery with time. This new threshold obtained by the QBlue tests is higher than those assessed by flow cytometry, where cell recovery was not taken into account. Hence, this highlights the need to use at least two techniques to study the effectiveness of a treatment. Going further, those techniques should be complementary in order to obtain a more valuable results. In this way, the data obtained from flow cytometric analysis can be perfectly complemented with different QBlue tests performed with time.

A clonogenic assay was performed after minibeam cell irradiation. Despite not showing the data due to the fact that this assay was not performed at least three times as the other techniques, the threshold dose obtained was 18-Gy valley dose for MBRT. This endpoint was the same that the one assessed previously by using the QBlue test.

In addition, Sarun et al. (paper in preparation) encountered that the valley dose threshold was around 20 Gy for a more radioresistant cell line (9L) irradiated with minibeams. This endpoint assessed by QBlue at 20 Gy agrees with those established around 18 Gy for the more radiosensitive F98 cell line [16,17].

## Conclusions

In our work we found a higher efficiency of MBRT in respect to BB for F98 cell-irradiation, mainly due to the large ratio of remaining early apoptotic cells, as well as a lower cell recovery than those obtained with a broad field.

Hence, we have demonstrated that considering only the ratio of dead cells (necrotic and late apoptotic cells) is not sufficient to assess the effectiveness of a radiation treatment, and parameters like the percentage of early apoptotic cells as well as the cellular recovery at several post-irradiation times should also be considered.

In addition, although further studies are needed, MBRT might offer probably a remarkable healthy tissue sparing in comparison to BB, as already observed in some first experiments [13].

## Competing interests

The authors declare that they have no competing interests.

## Authors' contributions

SG: participated in the design of the study, prepared the samples for irradiations, carried out all the post-irradiation techniques, performed the statistical analysis, and wrote the main part of the manuscript.

SS: participated in the coordination, helped preparing the samples for irradiations and with the performance of QBlue test, and helped to draft the manuscript.

AB: helped to draft the manuscript.

YP: carried out all the dose calculations and sample irradiations, participated in the design and coordination of the experiments and helped to draft the manuscript.

MS: participated in the design and coordination of the experiments and helped to draft the manuscript.

All authors read and approved the final manuscript.
